# A 5.8 Mb interstitial deletion on chromosome Xq21.1 in a boy with intellectual disability, cleft palate, hearing impairment and combined growth hormone deficiency

**DOI:** 10.1186/s12881-015-0220-z

**Published:** 2015-09-01

**Authors:** M. Giordano, C. Gertosio, S. Pagani, C. Meazza, I. Fusco, E. Bozzola, M. Bozzola

**Affiliations:** Laboratory of Genetics, Department of Health Sciences, University of Eastern Piedmont, Via Solaroli 17, 28100 Novara, Italy; Fondazione IRCCS Policlinico San Matteo, University of Pavia, Pavia, Italy; Department of Internal Medicine and Therapeutics, University of Pavia, Fondazione IRCCS Policlinico San Matteo, Pavia, Italy; Department of Pediatric Medicine, IRCCS Ospedale Pediatrico Bambino Gesù, Rome, Italy

**Keywords:** Xq21.1 deletion, Short stature, Pituitary hormone deficiency, Intellectual disability

## Abstract

**Background:**

Deletions of the long arm of chromosome X in males are a rare cause of X-linked intellectual disability. Here we describe a patient with an interstitial deletion of the Xq21.1 chromosome.

**Case presentation:**

In a 15 year boy, showing intellectual disability, short stature, hearing loss and dysmorphic facial features, a deletion at Xq21.1 was identified by array-CGH. This maternally inherited 5.8 Mb rearrangement encompasses 14 genes, including *BRWD3* (involved in X-linked intellectual disability), *TBX22* (a gene whose alterations have been related to the presence of cleft palate), *POU3F4* (mutated in X-linked deafness) and *ITM2A* (a gene involved in cartilage development).

**Conclusion:**

Correlation between the clinical findings and the function of gene mapping within the deleted region confirms the causative role of this microrearrangement in our patient and provides new insight into a gene possibly involved in short stature.

## Background

Cytogenetically detectable chromosomal deletions involving chromosome X are rare in males due to the incompatibility with life of nullisomy for large portions of this chromosome. In particular, deletions at Xq21 are poorly described in the literature and only a few cases are reported of male patients with complex phenotypes including neurosensorial deafness, choroideremia, intellectual disability and dysmorphisms [1]. About 50 % of the carrier females display almost or completely skewed X inactivation, silencing the abnormal X resulting in a relatively benign phenotype [2]. The development of high resolution genomic arrays has made it possible to increase the imbalance detection rate with the identification of cryptic small rearrangements on the X chromosome, some of these containing only a few genes or even just a single gene [3, 4]. However, despite the improvement of the analysis resolution, rearrangements at Xq21 are still rarely reported.

Here we describe a male patient with an interstitial deletion of the Xq21.1 chromosome, determined through array comparative genome hybridization (aCGH). We sought to explain the clinical outcome, according to genotype-phenotype correlation, focusing on the genes possibly involved in the clinical phenotype including intellectual impairment, hearing loss, cleft-palate and short stature.

## Case presentation

The patient was born at the 41st week of gestation by caesarean section due to fetal macrosomia. The birth weight was 4,530 g (>97° percentile), birth length 54 cm (90–97° percentile), and head circumference 40 cm (>97° percentile). Apgar scores were 9 and 10 at 1 and 5 minutes, respectively. The propositus was the only child of non-consanguineous Italian parents. The father was 36 and the mother was 28 years old. At birth, the patient showed cleft palate (plastic surgery of the hard palate was performed at the 5th month of life), hip dysplasia (corrected with retractor), right blepharoptosis (surgically corrected at 7 years of age) and dysmorphic face. At the fourth month of age, the parents noted the presence of severe head and limb hypotonia and a delayed psychomotor development. At 18 months, bilateral sensorineural hearing loss was diagnosed and a bilateral hearing aid was implanted.

The standard cytogenetic analysis revealed a normal male karyotype (46,XY). The patient proved negative for chromosome 22.11.2 deletion syndrome and fragile-X syndrome analysis. The family history was negative for intellectual disability.

Neurological examinations were performed, including a brain MRI at the age of 1 year with evidence of perinatal outcome suffering of the white matter, thinning of the corpus callosum and mild ventricular asymmetry. At 8 years of age, the brain MRI was repeated and showed the presence of mild ectasia of the perivascular spaces, soft signal hyperintensity of the periventricular white matter in the occipital region bilaterally as incomplete myelination.

At 3 years of age, because of short stature, a growth hormone (GH) stimulation test with arginine (GH peak: 5 ng/ml; n.v. > 10 ng/ml) and with glucagon (GH peak: 2.1 ng/ml; n.v. > 10 ng/ml) were performed and led to a diagnosis of complete growth hormone deficiency (GHD). GH replacement therapy was therefore started at the age of 4 years and stopped at the age of 9 years, for poor growth response. The patient did not return for further medical examination until the age of 15 years, when he was reassessed at our center for growth arrest associated with suspected hypogonadism (gonadal volume 2 cc bilaterally). His height was 151 cm (−2.23 SDS), BMI 26.97 kg/m^2^ (1.47 SDS). The gonadotropin evaluation tests performed with luteinizing-hormone-releasing hormone (LHRH) revealed deficit of luteinizing hormone (LH) and follicle-stimulating hormone (FSH) with a LH peak of 4.1 mU/ml (prepubertal value) and FSH peak of 7.3 mU/ml (prepubertal value). The stimulation test with growth-hormone-releasing hormone (GHRH) and arginine confirmed the severe GHD (GH peak: 0.1 ng/ml; n.v. > 19 ng/ml). Insulin-like growth factor (IGF-1) was below the normal range (<25.0 ng/ml; −7.81 SDS). Adrenal, thyroid, liver and kidney function showed normal range values. The ECG and the cardiological examination were normal. A delayed bone age of 2 years compared with chronological age was detected. A pituitary MRI was also performed and showed a normal morphology of the adenohypophysis, smaller than normal for the age without significant alterations in signal intensity before, during and after the administration of contrast medium. The patient therefore restarted GH replacement therapy.

For the psychomotor retardation with hyperactivity, the child attends psychomotor and speech therapy regularly and he is currently being treated with Levomepromazine (75 mg/day) and Promazine (50 gtt/day). The boy has a poor ability to tolerate frustration and there have been some episodes of verbal aggression against its peers. A neuropsychiatric test (Wisc IV test) showed an intellectual disability with an IQ < 70.

**Fig. 1:**
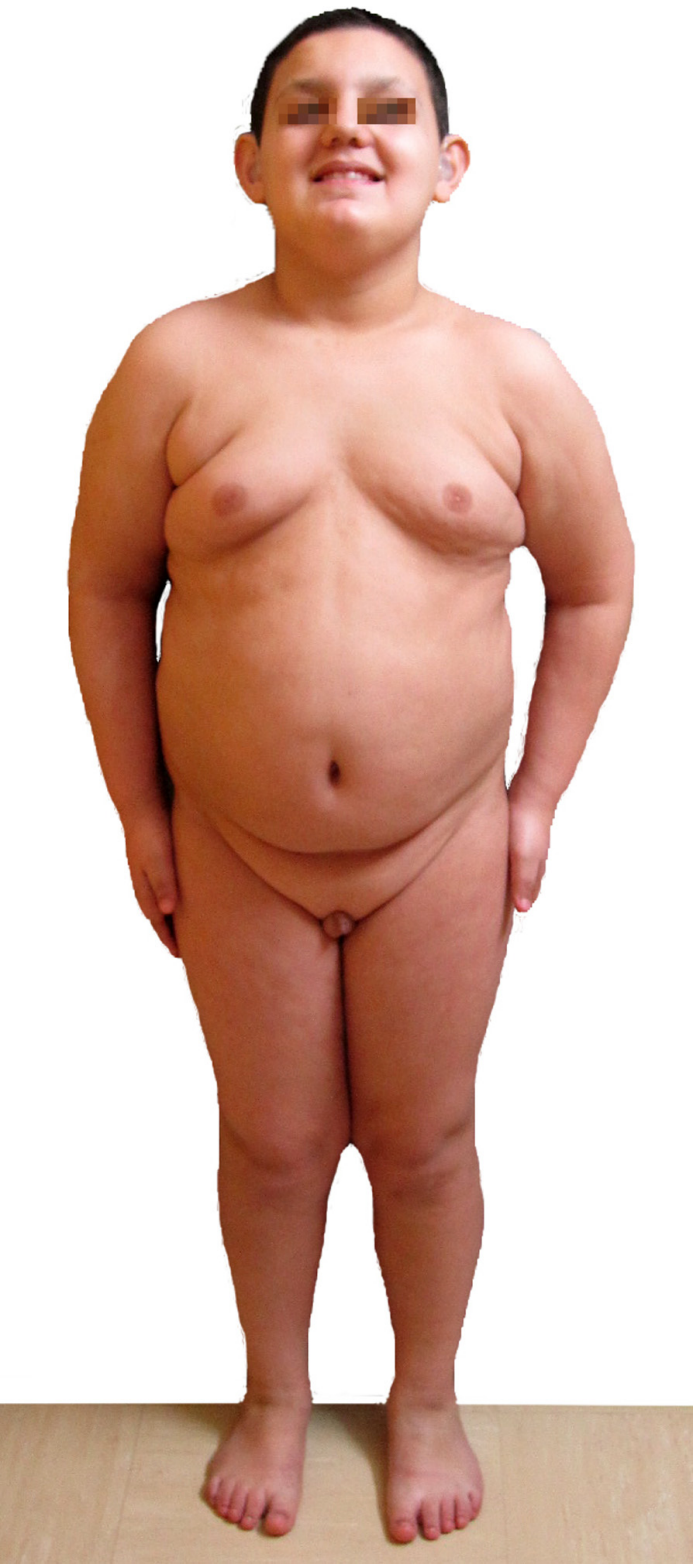
Morphological appearance of the patient at 15 years of age. Gynoid habitus, abdominal fat, pseudogynecomastia (presence of only adipose tissue), hypoplastic genitalia with microtestes (volume of 2 ml), generalized hypotonia, genus valgus, pes planus, short toes

**Fig. 2:**
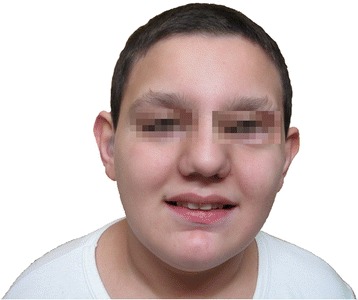
Facial features of the patient. Patient showing facial characteristics, including macrocephaly, broad forehead, prominent nose root and coarse nasal pyramid, anteverted nares, short palpebral fissures, large and prominent ears

**Fig. 3:**
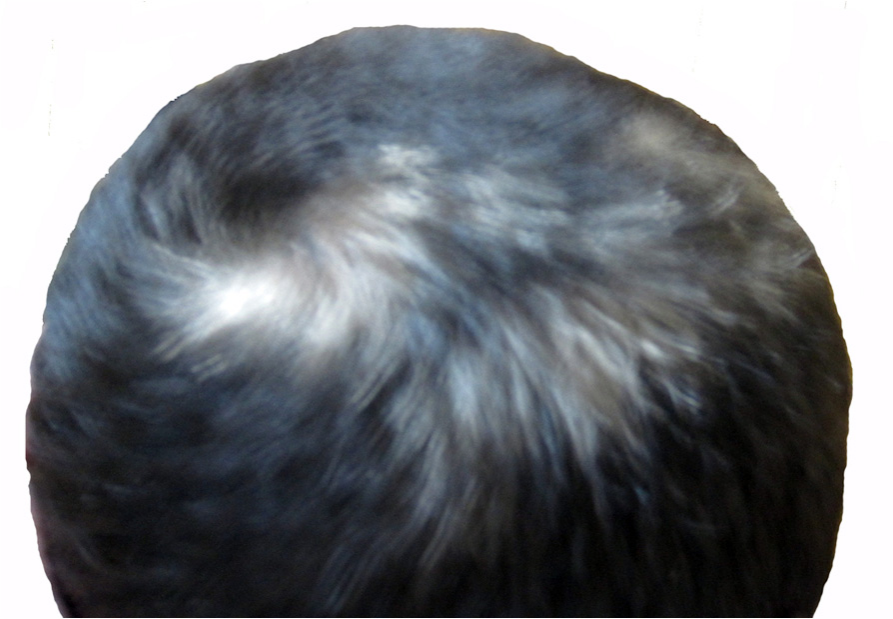
White tuft of hair on the top of the head

Concerning the morphological aspect (Fig. 1), the patient presents gynoid habitus, abdominal fat, pseudogynecomastia (presence of only adipose tissue), hypoplastic genitalia with microtestes (volume of 2 ml), generalized hypotonia, genus valgus, pes planus, short toes, café au lait spot on the left shoulder and in the right lumbar region. Facial features (Fig. 2) are characterized by: macrocephaly, broad forehead, prominent nose root and coarse nasal pyramid, anteverted nares, white tuft of hair on the top of his head (Fig. 3).

The mother of the boy presents some atypical characteristics, such as coarse facial features, prominent frontal bossing, big hands and progressive hearing impairment. She showed normal height and good intellectual development.

## Material and Methods

### Array CGH

DNA was extracted from whole blood of the patient by standard procedures. Array Comparative Genomic Hybridization (array-CGH) was performed using the Cytochip ISCA 4x180K (BlueGnome) containing 181,873 oligonucleotide probes (BlueGnome Ldt, Cambridge, UK) with a mean resolution of 16.30 Kb (25 Kb resolution on the backbone, 3.4 Kb resolution on genes). The CytoChip ISCA arrays are designed to investigate constitutional disorders through a combination of increased probe density in regions or genes associated with known constitutional disorders and regular spacing of probes on the genomic backbone, with no known disease association. Data analysis was performed using InnoScan 710 Microarray Scanner (Innopsys Inc. Chicago IL, USA) and Bluefuse software (BlueGnome Ldt, Cambridge, UK). Copy number variations (CNVs) reported in the Database of Genomic Variants http://projects.tcag.ca/variation/and in in-house databases of benign CNVs were excluded from further analysis. Genomic positions refer to the Human Genome February 2009 assembly (GRCh37/hg19).

### Sequencing

The *PROP1* and *GJB2* genes were screened for the presence of causative mutations by direct sequencing. Briefly genomic DNA was amplified by polymerase chain reaction (PCR) using primers designed to specifically amplify the coding regions and the intron/exon boundaries of each gene. PCR conditions and primer sequences are available upon request. The PCR products were visualized on a 2 % agarose gel and purified using ExoSAP-IT enzymatic PCR clean up system (Affimetrix, Santa Clara, CA). The purified products were then sequenced with the Big Dye Terminator kit (Applied Biosystems, Foster City, CA) and the automatic sequencer ABI PRISM 3100 Genetic Analyzer (Applied Biosystems, Foster City, CA).

### Database search

We performed a search in DECIPHER database (https://decipher.sanger.ac.uk/) and ISCA (https://iscaconsortium.org/) in order to identify cases of patients carrying similar deletions.

## Results

At the age of 18 months, the patient was tested for the presence of causative mutations in the gene encoding connexin 26 (*GJB2*), as he manifested bilateral neurosensorial hearing loss. No mutation associated with hearing impairment was identified in this gene.

**Fig. 4:**
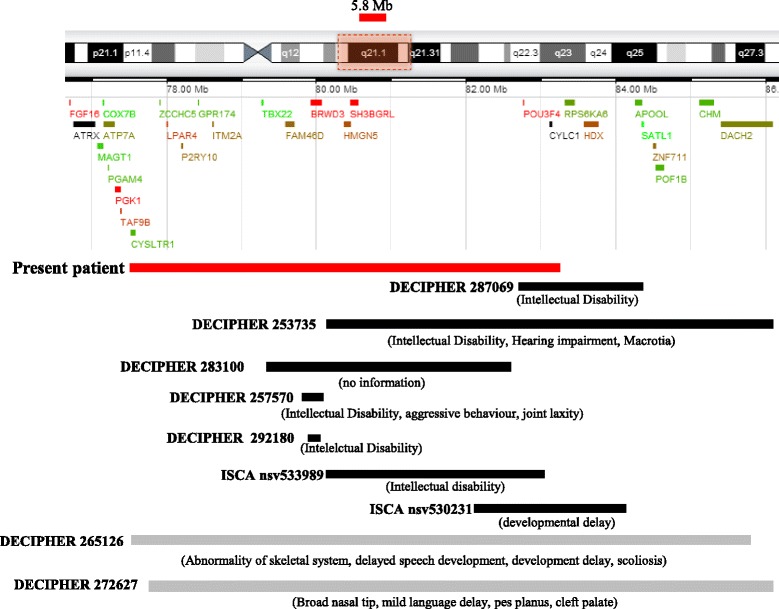
Xq21.1 Deletion. **a** Graphic representation of part of Xq (corresponding to the region included in the dotted box) with protein coding genes included in this region. The deletion at Xq21.1 detected in our patient (ChrX:77.456.818x1,77.489.632-83.255.802x0,83.287.869x1) is represented by a red rectangle above the boxed region. **b** Schematic representation of the Xq21.1 deleted region (*in red*) in our patient and other deletions reported in the Decipher and ISCA database in male patients (*in black*) and female patients (*in grey*). The ID of each patient is reported. The clinical features of the patients are reported in parentheses below the corresponding deletion as described in Decipher (https://decipher.sanger.ac.uk/) and ISCA (https://iscaconsortium.org/)

The array-CGH analysis, performed at the age of 13 years, revealed the presence of an interstitial deletion at Xq21.1 (77.456.818x1,77.489.632-83.255.802x0,83.287.869x1), of approximately 5.8 Mb (Fig. 4). The same deletion was present at the heterozygous state in the mother.

A paternal inherited deletion on chromosome 22q11.2 of about 105 kb (18.894.864x2, 18.905.139-19.010.478x1, 1.190.35352x2) was also identified, included within the 3 Mb deletion of the DiGeorge syndrome. However, it is likely a benign CNV as it is present in the Database of Genomic Variants (http://dgv.tcag.ca/dgv).

The Xq21.1 deleted region was examined using the Human resource websites (http://www.ncbi.nlm.nih.gov/projects/genome/guide/human) at NCBI and the Archive EnsEMBL (http://www.ensembl.org/info/website/archives/index). This region contains 14 genes, some of them with a known function (Table 1). Among these, three genes (*TBX22, BRW3* and *POU3F4*) have been previously reported mutated in well defined disorders, thus facilitating the phenotype-genotype correlation in our patient (Table 1). However, none of the genes included in the deletion have been previously correlated to the pituitary hormone deficiencies observed in our patient. The *PROP1* gene was thus sequenced in order to detect a gene defect elsewhere that could explain the GH and gonadotropin deficiency, but no mutation was identified.

**Table 1:** Protein encoding genes with a known function included in the Xq21.1 deletion

Gene	Protein	Protein function	Disease
*CYSLTR1*	Cysteinyl leukotriene receptor 1	Mediation of bronchoconstriction via activation of a phosphatidylinositol-calcium second messenger system	None
*ZCCHC5*	Zinc finger, CCHC domain containing 5	Member of a family of gag-related retrotransposon genes	None
*LPAR4*	Lysophosphatidic acid receptor 4	Monocytic differentiation	None
*P2RY10*	Purinergic receptor P2Y, G-protein coupled, 10	Stimulation of diacylglyceride-dependent protein kinases	None
*GPR174*	G protein-coupled receptor 174	Lysophosphatidylserine receptor involved in intracellular cAMP increase	None
*ITM2A*	Integral membrane protein 2A	Chondrogenesis	None
*TBX22*	T-box 22	Palatogenesis	Cleft palate with ankyloglossia
*BRWD3*	Bromodomain and WD repeat domain containing 3	Chromatin-modification	Intellectual disability X-linked with macrocephaly
*HMGN5*	High mobility group nucleosome binding domain 5	Nucleosomal binding and transcriptional activation	None
*SH3BGRL*	SH3 domain binding glutamate-rich protein like	Signal transduction	None
*POU3F4*	POU class 3 homeobox 4	Inner ear development	Non-syndromic hearing loss
*CYLC1*	Cylicin, basic protein of sperm head cytoskeleton 1	Spermatid differentiation	None

## Discussion

We report a maternally inherited deletion of approximately 5.8 Mb at Xq21.1, in a male subject with developmental delay, dysmorphic facial features, cleft palate, intellectual disability, short stature and hearing loss. The deleted region includes 14 OMIM genes: *CYSLTR1, ZCCHC5, LPAR4, P2RY10, GPR174, ITM2A, TBX22, BRWD3, HMGN5, SH3BGRL, HMGN5, SH3BGRL, POU3F4, CYLC1* (Fig. 4 and Table 1)*.* Among these, *TBX22, BRWD3* and *POU3F4* well explain some of the clinical features of this patient.

Mutations in the *TBX22* gene are a well established cause of X-linked cleft palate with ankyloglossia as well as contributing to the prevalence of isolated cleft palate [5]. More rarely, other craniofacial anomalies including cleft lip and hypodontia have also been related to *TBX22* variants [6]. The phenotypic spectrum of subjects bearing *TBX22* mutations can vary, even within the same family, from asymptomatic females to males or females with a bifid uvula, a cleft of the soft palate, or a complete cleft of the hard and soft secondary palate, along with ankyloglossia [7].

Genetic hearing loss has an extremely varied etiology, with a plethora of genes involved in the autosomal and X-linked forms with overlapping phenotypes [8]. Among the main genetic defects, mutations within connexin 26 (*GJB2*) and connexin 30 (*GJB6*) are usually associated with severe to profound sensorineural deafness. Our patient had been screened for the presence of mutations in *GJB2* before performing array-CGH, and was negative. The deafness causative gene is undoubtedly *POU3F4,* which causes the X-linked neurosensorial deafness *DFN3*. This defect is associated with either *POU3F4* point mutations or small deletions in a region located at 900 Kb upstream of the gene and disturbing a regulatory element [9]. Male patients show a well characterized phenotype with both stapes fixation and progressive mixed hearing loss [10, 11]. Hearing loss was also observed in the mother of our patient as well as in about 40 % of the *POU3F4* mutation carrier females. Carrier females usually exhibit a postlingual onset of the hearing impairment that progresses over time [12] with a variable expressivity attributable to variations in the degree of skewing of X inactivation.

Loss of function mutations affecting *BRWD3* are associated with a phenotype including mild to moderate intellectual disability, macrocephaly, dysmorphic facial features, skeletal signs and behavioral disturbance [13, 14]. Among these alterations, a partial deletion encompassing 74 Kb and including the 30 last exons of the *BRWD3* gene was reported in a male with high forehead, deep-set eyes, hypertelorism, short palpebral fissures, anteverted nares, downturned corners of the mouth, pointed chin, and skeletal anomalies. This phenotype is similar to that of the patient presented here: macrocephaly, dysmorphic facial features including prominent forehead and abnormal ears, behavioral disturbance, skeletal symptoms like pes planus, and cubitus valgus. These findings strongly suggest that the lack of *BRWD3* causes intellectual disability, macrocephaly and possibly the skeletal symptoms (pes planus and cubitus valgus) observed in our patient. It has been demonstrated that BRWD3 plays a crucial role in ubiquitination, as part of the ubiquitin/proteasome system. In Drosophila, BRWD3 belongs to the CUL4-ROC1-DDB1 E3 ligase complex in which it acts as a CULLIN (CUL)4-associated factor that mediates light-dependent binding of CRY (Cryptochrome, a circadian photoreceptor) to the complex, inducing the ubiquitination of dCRY and its light-induced degradation [15]. The ubiquitin-proteasome system plays a crucial role in brain development and is a critical regulator of the synaptic plasticity and long-term memory formation [16]. Intriguingly, in humans mutations of the CUL4B, which encode a ubiquitin 3 ligase subunit cause an X-linked syndrome characterized by intellectual impairment, macrocephaly, central obesity, hypogonadism, pes cavus and tremor [17], a phenotype that largely overlaps with that observed in patients carrying BRWD3 mutations/deletions, including the present case. As both BRWD3 and CUL4B are part of the same complex it is likely that alterations of BRWD3 influence the ubiquitin-proteasome system similarly to other intellectual disability syndromes, whose prototype is represented by the Angelman Syndrome which is caused by mutations of the ubiquitin ligase-encoding UBE3A gene (MIM 105830).

The search in the Decipher and ISCA databases for similar size deletions at Xq21.1 led to the identification of six deletions in males with syndromic intellectual disability and developmental delay (DECIPHER 287069,253735, 257570,292180 and ISCA nsv533989, nsv530231, Fig. 4) and in one patient with no further phenotype details (DECIPHER 283100, Fig. 4) but none of these exactly corresponds to that of our patient. However, among those observed in female patients, there are two deletions (DECIPHER 262726 and 265126, Fig. 4) that completely cover that of our patient. The milder associated phenotype includes broad nasal tip, skeletal defects, speech delay, and cleft palate, which are also features of the present patient.

Other remarkable clinical signs of our patient are short stature and multiple pituitary hormone deficiency. The presence of mutations in *PROP1*, the most common genetic cause of combined pituitary hormone deficiency (CPHD) associated with short stature [18], was excluded. It is interesting that the deletion partially overlaps with regions duplicated in patients with multiple congenital anomalies, developmental delay, GH deficiency and short stature [19–21]. This suggests that Xq21.1 might contain either a protein coding gene or a regulatory element involved in pituitary hormone secretion. Within the 5.8 Mb region there is no evidence for a protein coding gene directly involved in pituitary functioning. However, *ITM2A*, included in the deletion, might be related to the severe short stature as it encodes an integral transmembrane protein involved in early cartilage development [22]. It has been suggested that the expression of *ITM2A* influences the chondrogenic differentiation potential of mesenchymal stem cells in vitro [23]. It might be hypothesized that the absence of ITM2A greatly influences the cartilage development with a possible impact on postnatal growth.

## Conclusions

In conclusion, a detailed comparison of the clinical characteristics and the function of the genes included in the Xq21.1 delete region confirm the causative role of this rearrangement in our patient. Moreover, the presence of a gene involved in cartilage differentiation suggests that *ITM2A* might be responsible for growth impairment.

## Consent

Written informed consent was obtained from the mother of the patient for publication of this Case report and any accompanying images. A copy of the written consent is available for review by the Editor of this journal.

## Abbreviations

array-CGH: Array comparative genomic hybridization
XLID: X-linked Intellectual disability
GHD: Growth hormone deficiency
MRI: Magnetic resonance imaging
IQ: Intelligence quotient
GH: Growth hormone
LHRH: Luteinizing-hormone-releasing hormone
LH: Luteinizing hormone
FSH: Follicle-stimulating hormone
GHRH: Growth-hormone-releasing hormone
IGF-1: Insulin-like growth factor
SDS: Standard deviation score
ECG: Electrocardiography
CNVs: Copy number variations
PCR: Polymerase chain reaction
CPHD: Combined pituitary hormone deficiency
